# To Lie or Not to Lie: The Views of People With Dementia and Their Carers

**DOI:** 10.1093/geroni/igaa057.2734

**Published:** 2020-12-16

**Authors:** Dympna Casey, Una Lynch, Adeline Cooney, Catherine Houghton, Siobhan Smyth, Fionnuala Jordan, Heike Felzman

**Affiliations:** 1 National University of Ireland Galway, Galway, Ireland; 2 Sonista, Banbridge, Northern Ireland, United Kingdom; 3 Maynooth University, Maynooth, Galway, Ireland; 4 National University of Ireland Galway, Galway, Galway, Ireland; 5 School of Nursing & Midwifery, National University of Ireland Galway, Galway, Galway, Ireland; 6 School of Humanities, National University of Ireland Galway, Galway, Galway, Ireland

## Abstract

In Ireland over 36,000 people with dementia live at home cared for by informal carers. Yet often these carers do not know how to deal with cognitive symptoms, including repeated questions wherein ‘truthful’responses cause distress. Carers face a dilemma, do they avoid, distract or ‘correct’ the person and tell the ‘truth’, or lie? This paper explores the concept of lying from the perspective of the carer and person with dementia. A descriptive qualitative methodology was used. Focus groups with a purposive sample of people with memory problems (n = 14) and carers (n = 18) were conducted. The results found that deliberate lying with the intention to deceive was deemed unacceptable by all. However, in certain circumstances telling a ‘good lie’ or ‘white lie’ was considered acceptable when the carer knew the person and the intention behind the ‘lie’ was to mitigate the distress of the person with dementia.

